# Association of Serum Uric Acid Levels in Psoriasis: A Systematic Review and Meta-Analysis

**DOI:** 10.1097/MD.0000000000003676

**Published:** 2016-05-13

**Authors:** Xin Li, Xiao Miao, Hongshen Wang, Yifei Wang, Fulun Li, Qiong Yang, Rutao Cui, Bin Li

**Affiliations:** From the Department of Dermatology (XL, XM, YW, FL, BL), Yueyang Hospital of Integrated Traditional Chinese and Western Medicine, Shanghai University of Traditional Chinese Medicine, Shanghai, China; Department of Pharmacology and Experimental Therapeutics (XL, HW, RC), Boston University School of Medicine; and Department of Statistics (QY), Boston University School of Public Health, Boston, MA.

## Abstract

High levels of serum uric acid (SUAC) are frequently detected in patients with psoriasis. However, the relationship between psoriasis and hyperuricemia remains unknown. Here we conducted a meta-analysis to identify the SUAC levels in subjects with psoriasis and to determine whether there is an associated risk between psoriasis and hyperuricemia.

A comprehensive search of the literature from January 1980 to November 2014 across 7 databases (MEDLINE, Embase, Cochrane Central Register, and 4 Chinese databases) was conducted to determine whether there is an associated risk between psoriasis and hyperuricemia.

Among the 170 identified reports, 14 observational studies were included in this meta-analysis. We found a significant higher SUAC level (MD 0.68, 95% CI 0.26–1.09; *P* = 0.002) in patients with psoriasis in Western Europe, but no significant differences were found between the East Asia and India subgroup (MD 1.22, 95% CI –0.13–2.56; *P* = 0.08) or the Middle East subgroup (MD 0.48, 95% CI –0.49–1.44; *P* = 0.33). Similar results were obtained from the meta-analysis of SUAC levels in subjects with severe psoriasis.

Our meta-analysis showed that the correlation between psoriasis and hyperuricemia was either ethnicity- or region-dependent and that patients with psoriasis in Western Europe were more likely to have hyperuricemia.

## INTRODUCTION

Psoriasis is a chronic immune-mediated skin disease that affects ∼2% to 4% of the population in Western countries.^1^ Multiple types of psoriasis have been described, including pustular, guttate, and erythrodermic psoriasis, in addition to rarer forms such as psoriatic arthritis. In addition, patients with psoriasis may present itchy or painful lesions that negatively affect their quality of life. The pathogenesis of psoriasis is not completely understood; however, abnormal differentiation of keratinocytes and infiltration of inflammatory cells have been suggested.^2^ Inflammatory cytokines that are found in the psoriatic lesions have also been implicated.^3^ Patients with psoriasis are at greater risk of cardiovascular disease (CVD),^4^ metabolic syndrome (MetS),^5,6^ type 2 diabetes mellitus, dyslipidemia, hypertension, and obesity.^7,8^

Hyperuricemia has been detected more frequently in patients with psoriasis and has also been associated with CVD and MetS. Specifically, the prevalence of CVD has been linked with higher levels of uric acid (UA).^9,10^ In addition, hyperuricemia has been reported to cause adverse cardiovascular outcomes, especially sudden cardiac death.^11^ In 1958, Walkerin first suggested that psoriasis may be associated with hyperuricemia.^12^ Since then, several observational studies have reported conflicting results on the correlation between serum UA (SUAC) levels and psoriasis. A previous study observed higher SUAC levels in psoriasis patients, among 76 subjects with psoriasis and 68 persons without psoriasis from the United States in 1961.^13^ Moreover, the results of a larger prospective study indicated that psoriasis patients with extensive involvement of the skin tended to have a higher incidence of hyperuricemia.^14^ However, a cohort study conducted in England provided a conflicting result: no significant difference was found between the SUAC levels of a psoriasis group and a group composed of hospital employees (each with 41 subjects).^15^

Herein, we performed a meta-analysis of studies investigating the correlation of SUAC levels with psoriasis and evaluated the influence of potential confounding factors presently under discussion during the past 30 years. We hypothesized that the observed correlation between hyperuricemia and psoriasis might be dependent on the ethnicities of the patients involved or on the geographic locations of the studies.

## MATERIALS AND METHODS

### Data Sources and Searches

To identify relevant psoriasis studies that measured SUAC levels and having hyperuricemia as an outcome measure, 3 reviewers (XL, XM, and HSW) systematically searched MEDLINE, Embase, the Cochrane Central Register, the China National Knowledge Infrastructure database (CNKI), the Chinese Scientific Journals Full Text Database (CQVIP), the Wanfang Data Knowledge Service Platform, and the Chinese Biomedical Literature Service System (SinoMed) using the search terms: psoriasis, hyperuricemia, and uric acid. Papers published in English or Chinese and dated from January 1980 to November 2014 were included in this study.

### Study Selection

**FIGURE 1:**
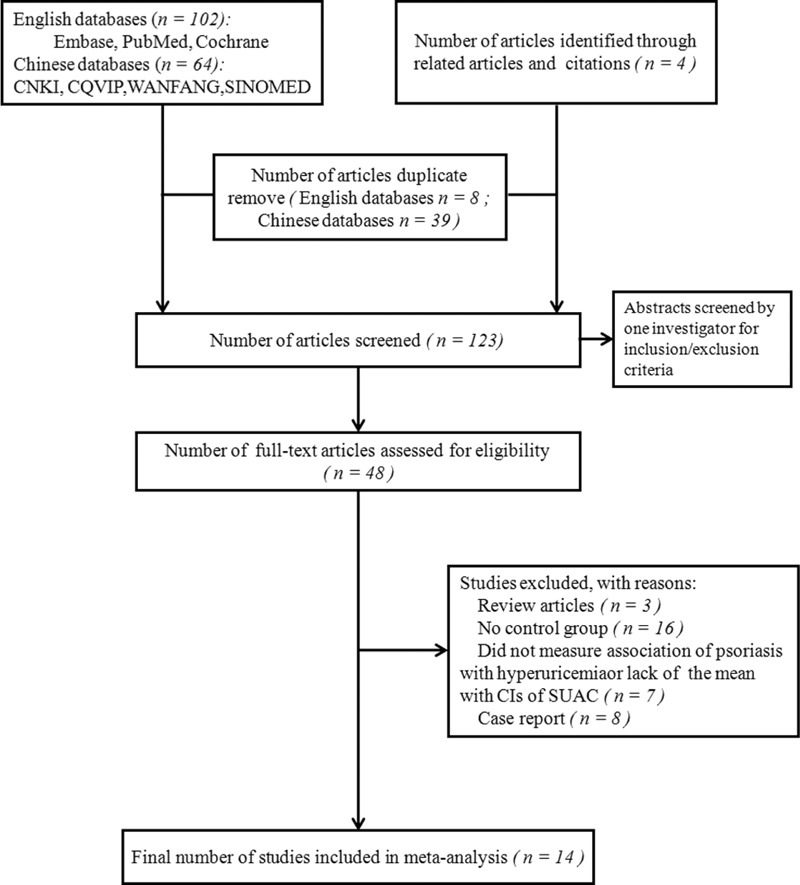
Literature search and study selection flowchart.

To determine eligibility for inclusion in the review, we screened abstracts for the following criteria: case-control, cross-sectional, cohort or nested case-control design; examination of hyperuricemia in relation to psoriasis; or analyses that compared the SUAC profiles of patients with psoriasis with those of control groups. There were no limitations on the participant's age, gender, or nationality. The selection criteria for inclusion were as follows: (i) human-only studies; (ii) availability of original data; (iii) availability of a control group; (iv) odds ratio (OR), risk ratio (RR), hazard ratio estimates with confidence intervals(s) (CI) (including enough data to calculate them); or hyperuricemia considered as a specific outcome event, or the mean with CIs of SUAC provided. In this study, we identified 166 articles from the initial search (Figure 1). Through manual review of the citations from these articles, we found 4 additional articles. After removing 47 duplicate articles and reading the 123 individual abstracts, we identified 48 original studies that potentially satisfied the selection criteria. After reviewing the full text of these 48 studies, we excluded 34 articles because of study type (reviews or case reports) or methodology (lack of control group, no measure of correlation between psoriasis and hyperuricemia, no measurement of confidence interval around the mean SUAC levels). Finally, we selected 14 studies that met the inclusion criteria for this systematic review.^15–28^ A flowchart of the search process is shown in Figure 1.

### Data Extraction and Quality Assessment

Three reviewers independently collected descriptive data for each included study: (i) the first author; (ii) study characteristics (i.e., year, duration, country, setting, and design); (iii) characteristics of participants (i.e., mean age, number of cases and control subjects, number of cases receiving systemic therapy for psoriasis, and number of subjects using acid-lowering drugs); and (iv) outcome characteristics (i.e., the RR of psoriasis associated with hyperuricemia along with the corresponding 95% CI, whether results were a primary analysis of the study or adjusted for comorbidities).

The Newcastle–Ottawa Scale^29^ was used to assess the study quality. This scale is categorized into 3 categories including selection, comparability, and exposure for case-control studies; and selection, comparability, and outcome for cohort studies. The selection category contains 4 quality items, the comparability contains 1 item, and the exposure contains 3.

### Data Synthesis and Analysis

The primary outcomes included the difference in mean SUAC levels between healthy controls and patients with psoriasis, as well as the correlation of psoriasis with the risk of hyperuricemia for each study. The degree of heterogeneity between studies was assessed using Cochrane *χ*^2^ and *I*^2^ tests. *P* value <0.10 or *I*^2^ value >50% is considered as substantial heterogeneity. In this case, DerSimonian and Laird random-effect models were considered in order to compute the global MD and RR. For *P* values >0.10 or *I*^2^ <50%, the between-study heterogeneity was not considered substantial and the fixed-effect models were used. To investigate possible reasons for heterogeneity, we performed subgroup analysis and meta-regression using prespecified variables and random-effects meta-analysis. Prespecified sources of heterogeneity in meta-regression included source population, study design, study quality, severity of psoriasis, presence of psoriatic arthritis, outcome ascertainment, and analysis of outcome. The methods and findings of the present review have been reported following the Meta-analysis of Observational Studies in Epidemiology (MOOSE) group guidelines and checklist.^30^ Review Manager 5.2 was used for meta-analysis (http://ims.cochrane.org/revman). Meta-regression was performed using STATA version 10.0 (STATA Corp, College Station, TX).

This study used published data and thus ethical approval was not necessary.

## RESULTS

### General Characteristics and Methodology Assessment

Fourteen studies (Table 1) were selected out of 170 identified from the systematic review and were included in our meta-analysis (see Methods section for the selection process). Our meta-analysis consisted of 2 parts according to the type of response variable. The first part included 13 studies with continuous SUAC as the response variable and a total of 29,037 participants (1644 patients with psoriasis and 27,393 controls). The second part included 2 studies^18,26^ with dichotomous hyperuricemia as the response variable and a total of 617 participants (265 patients with psoriasis and 352 controls) who met the inclusion criteria for the dichotomous variables of the systematic review. Of these14 studies, 12 were cohort studies, 1 was a case-control study, and 1 was a cross-sectional study (Table 1). In addition, 13 of these studies provided means with CIs of SUAC levels of the psoriasis and control groups. Two studies considered hyperuricemia as a specific outcome event and provided RR. Eight studies reported a statistically significant difference in SUAC levels between psoriasis patients and controls. Five of the studies were conducted among outpatients with psoriasis. Studies were conducted in diverse locations, including Western Europe; East Asia and India; and the Middle East, among which half of the countries (7) belongs to Asia.

**TABLE 1:**
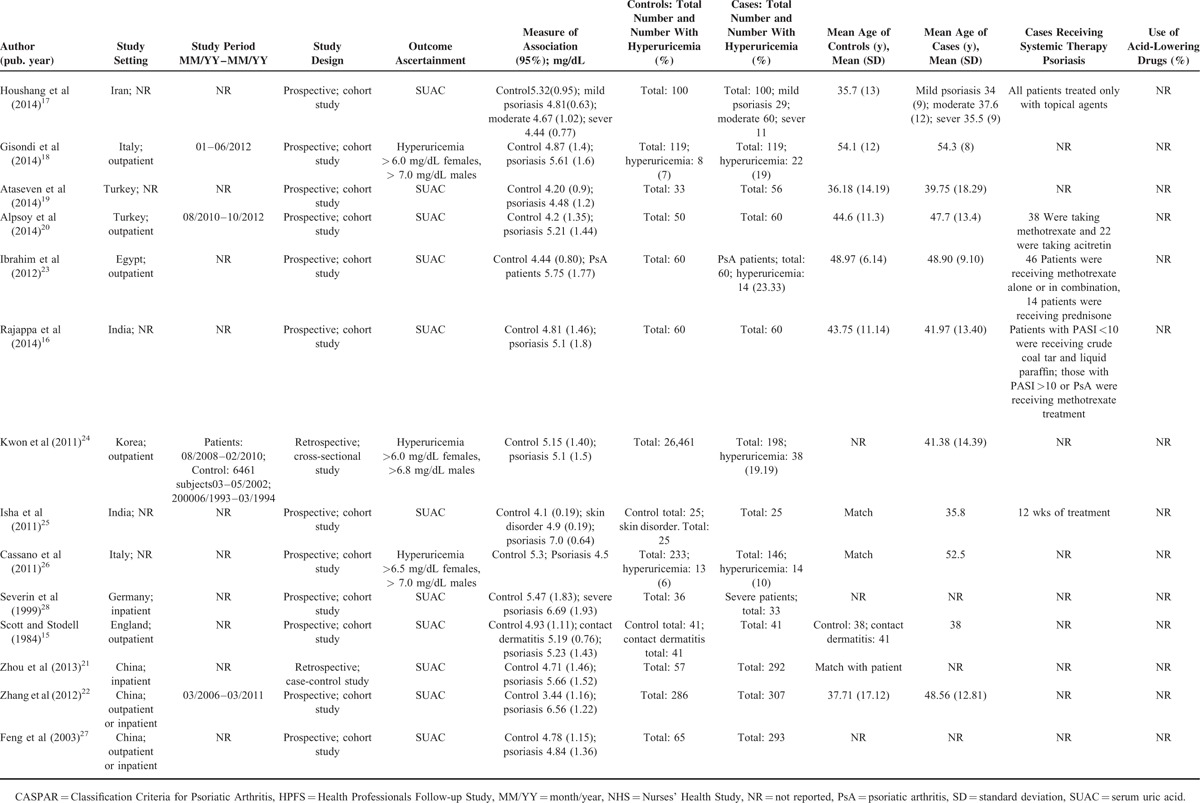
Observational Studies Included in the Meta-Analysis

### Score of Included Studies

The Newcastle–Ottawa Scale scores obtained ranged from 4 to 9 as shown in Table 2. Specifically, 12 of the studies were deemed of medium-quality (4–6 stars) and 2 were high-quality studies (7 or >7 stars).

**TABLE 2:**
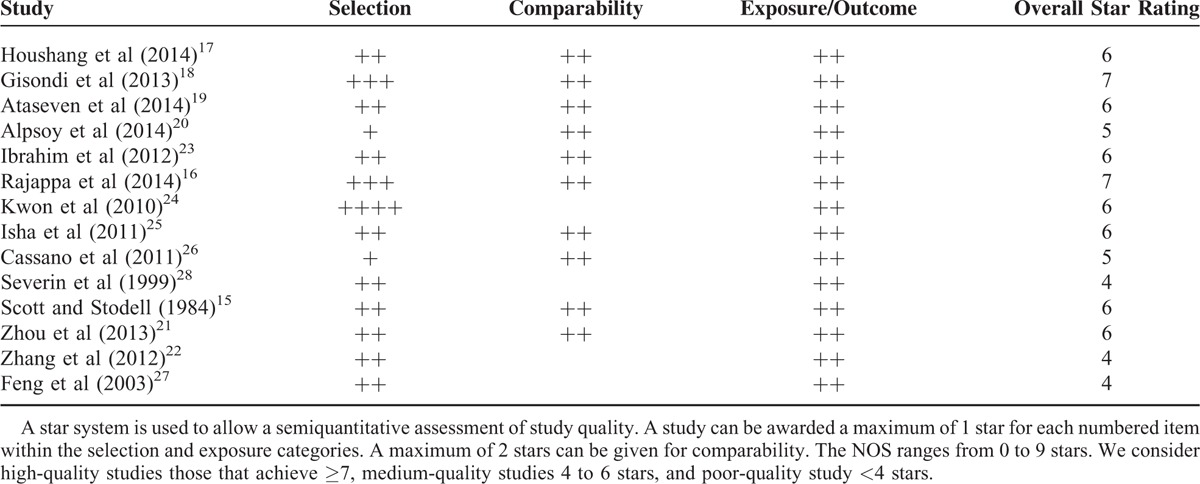
Newcastle–Ottawa Scale (NOS) Quality Assessment Table

### Primary Outcomes (Continuous Variables)

**FIGURE 2:**
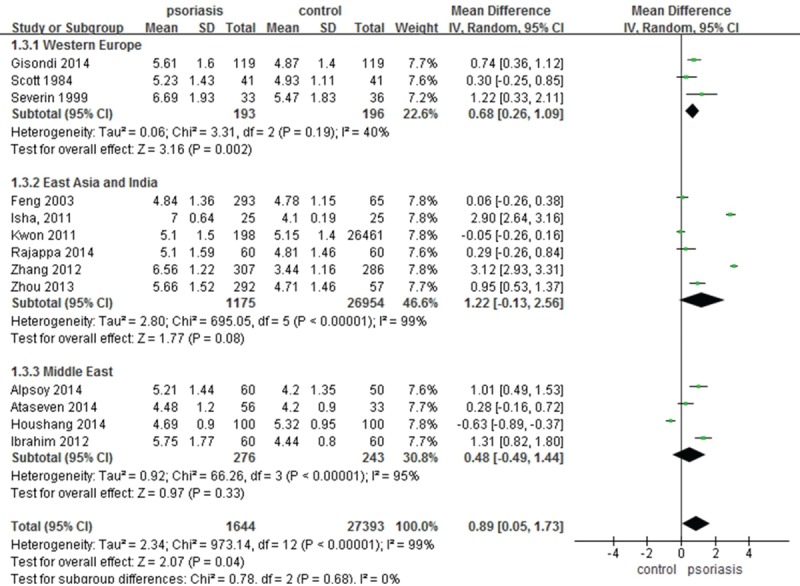
Meta-analysis of the impact on (SUAC of patients with psoriasis compared with controls. The MD in SUAC levels of patients with psoriasis compared with controls. The point estimate (center of each green square) and statistical size (proportional area of the square) are represented. Horizontal lines indicate 95% confidence intervals. The subtotal and total MD (diamond) were calculated using a random-effects model. MD = mean difference, SUAC = serum uric acid concentration.

The meta-analysis of SUAC levels of subjects with psoriasis revealed significant between-study heterogeneity (*I*^2^ = 99%, *P* <0.00001). In addition, there was heterogeneity within each separate subgroup (Western Europe *I*^2^ = 40%, *P* = 0.19; East Asia and India *I*^2^ = 99%, *P* < 0.00001; and Middle East *I*^2^ = 95%, *P* <0.00001). With random-effects modeling, the pooled data for 13 studies showed a significant difference in SUAC levels between psoriasis patients and controls (MD 0.89, 95% CI 0.05–1.73; *P* = 0.04). Although heterogeneity was high, subgroup analyses was able to identify an optimal subgroup in Western Europe (MD 0.68, 95% CI 0.26–1.09; *P* = 0.002). On the contrary, no significant differences were found between the subgroups from East Asia and India (MD 1.22, 95% CI –0.13–2.56; *P* = 0.08) or the Middle East (MD 0.48, 95% CI –0.49–1.44; *P* = 0.33) (Figure 2). Meta-regression of SUAC levels of patients with psoriasis compared with controls did not reveal any statistically significant sources of heterogeneity (Table 3).

**TABLE 3:**
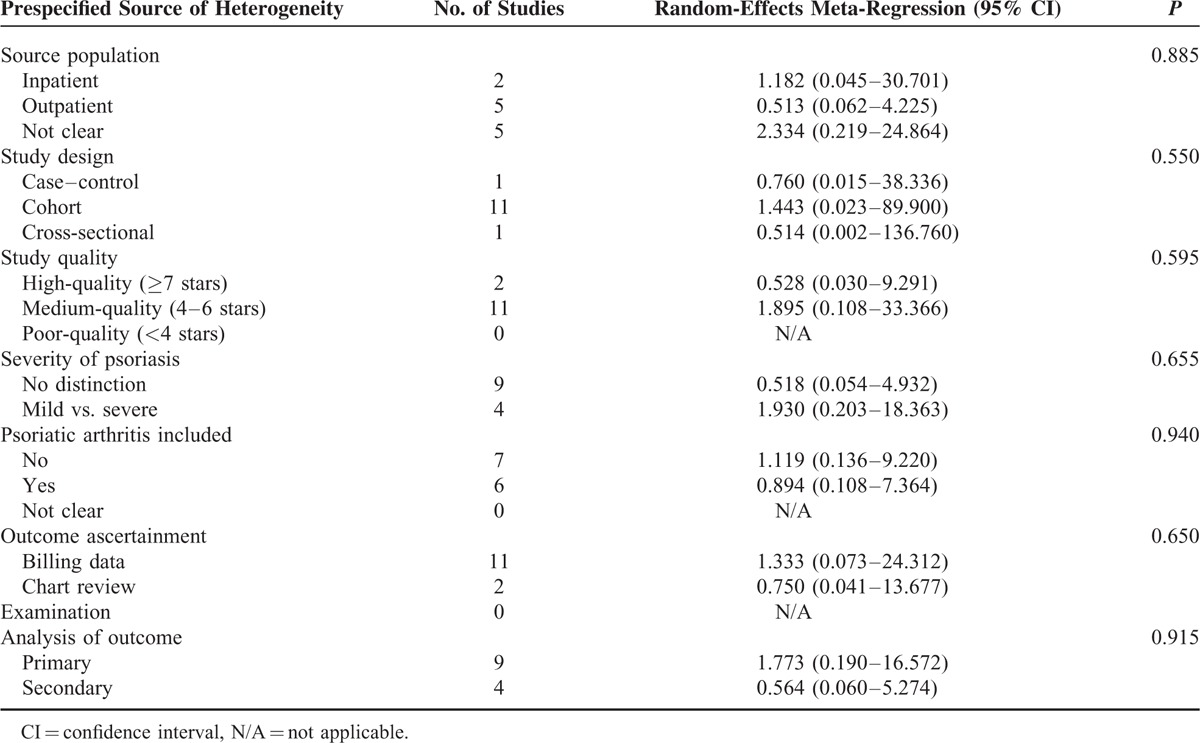
Potential Prespecified Sources of Heterogeneity Explored Among Studies Reporting SUAC Levels of Patients With Psoriasis Compared With Controls

### Secondary Outcomes (Data for Severe Psoriasis and Italian)

**FIGURE 3:**
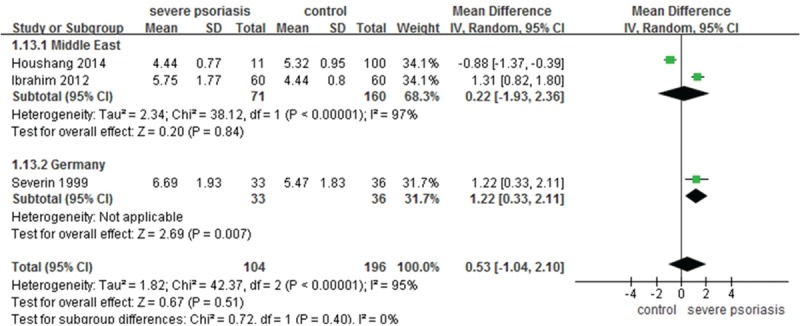
Meta-analysis of the impact on SUAC of patients with severe psoriasis compared to controls. The MD in SUAC levels of patients with severe psoriasis compared to controls. The point estimate (center of each green square) and statistical size (proportional area of the square) are represented. Horizontal lines indicate 95% confidence intervals. The subtotal and total MD (diamond) were calculated using a random-effects model. MD = mean difference, SUAC = serum uric acid concentration.

To identify the SUAC level in subjects with severe psoriasis, a meta-analysis with significant between-study heterogeneity (*I*^2^ = 95%, *P* <0.00001) was conducted. The pooled MD from the random-effects analysis for 3 studies was determined to be 0.53 (95% CI –1.04–2.10; *P* = 0.51). There was heterogeneity in the Middle East subgroup (*I*^2^ = 97%, *P* <0.00001), and no significant differences were found (MD 0.22, 95% CI –1.93–2.36; *P* = 0.84) (Figure 3). Overall, the significant difference in SUAC levels between psoriasis patients and normal controls in the Western Europe is likely to be clinically relevant and unlikely to be an artifact resulting from between-study heterogeneity.

**FIGURE 4:**
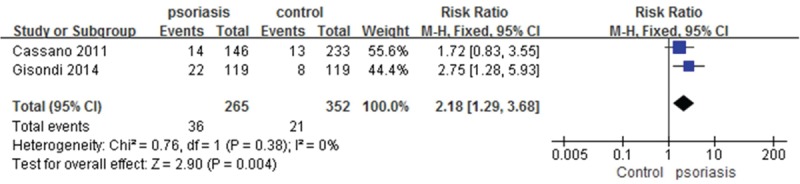
Meta-analysis of the prevalence of hyperuricemia in patients with psoriasis compared to controls in Italy. RR for psoriasis in subjects with hyperuricemia compared with total subjects. The point estimate (center of each blue square) and statistical size (proportional area of the square) are represented. Horizontal lines indicate 95% confidence intervals. The pooled RR (diamond) was calculated using a fixed-effects model. RR = relative risk.

Meta-analysis of hyperuricemia prevalence among patients with psoriasis and controls in Italy revealed low between-study heterogeneity (*I*^2^ = 0%, *P* = 0.38). With fixed effects modeling, we observed significant differences in hyperuricemia occurrences between psoriasis patients and controls in the 2 studies from Italy, with a pooled RR of 2.18 (95% CI, 1.29–3.68; *P* = 0.004) (Figure 4).

## DISCUSSION

The results from our meta-analysis suggest that the correlation between psoriasis and hyperuricemia might be either ethnicity- or region-dependent. Specifically, patients from Western Europe are more likely to have hyperuricemia, whereas our meta-analysis did not observe a significant correlation between psoriasis and hyperuricemia in East Asia and India or the Middle East.

The prevalence and incidence of psoriasis showed clear ethnic and geographic patterns. Psoriasis is more common in patients from the colder north than in patients from the tropics. The prevalence of psoriasis is the highest in Europe (0.6%–6.5%) and the United States (3.15%), and the lowest in West Africa (0.08%–0.9%) and East Asia (0.05%–1.23%), and absent in the Andean region of South America. The etiology of psoriasis is dependent on both genetic and environmental factors.^31^ The genetic component has been demonstrated in epidemiologic studies.^32^ Different lifestyles, eating, and social behaviors might also contribute to disease prevalence.^33^ Therefore, it is not surprising that the correlation between psoriasis and hyperuricemia displays an ethnicity and/or region-dependent pattern.

This meta-analysis combined data from 14 studies consisting of about total 29,416 participants. These studies include almost all the available epidemiological evidence worldwide on the topic. Although the correlation between elevated SUAC levels or hyperuricemia and psoriasis had been reported previously,^18,20–23,25,28,34^ we found a ethnicity/region-dependent pattern for this correlation. Specifically, the MD value 0 is outside the 95% CI of the SUAC levels in the Western Europe subgroup but within that of the East Asia and India or the Middle East subgroup. Similar results were obtained from the meta-analysis of the SUAC levels in subjects with severe psoriasis.

The 2 Italian studies^18,26^ included in the second part of the meta-analysis showed that the pooled risk of hyperuricemia was 2 times higher in psoriasis patients. In addition, elevated SUAC levels or hyperuricemia, even within the high-normal range, are positively associated with higher CVD risk of mortality,^35–38^ as well as MetS and its components.^35,39–41^ Together, these results suggest that the normal SUAC reference ranges used in the clinic might be inadequate for detecting early metabolic disturbance and CVD risk of mortality.

Severe psoriasis has been suggested to be an independent cardiovascular risk factor.^42^ Our meta-analysis suggests that UA may be a potential target for preventing the incidence of metabolic disturbances (insulin sensitivity, rises in blood pressure, and cholesterol)^39^ or cardiovascular risk factors.^35^ On the contrary, it remains to be determined whether the relationship between UA and cardiovascular events is circumstantial or causal in patients with psoriasis from Western Europe.

In any observational study, there is always a potential limitation for unknown confounders influencing the results. Our analysis has some limitations as well. For example, only 2 eligible observation studies from Italy were reviewed in the second part. In addition, the 4 studies from India and the Middle East included patients who were receiving systemic therapy, which may have reduced SUAC levels.^25^ Furthermore, high degree of heterogeneity is present in the East Asia and India or the Middle East subgroups. Therefore, any interpretation of the results must be done cautiously given these limitations.

To identify potential confronting factors for high heterogeneity, we assessed the effects of multiple variables on the findings among the included studies by meta-regression analysis. These variables include source population, study design, study quality, severity of psoriasis, psoriatic arthritis, outcome ascertainment, and analysis of outcome. We found no statistically significant difference in outcomes resulting from each of these variables.

Taken together, our meta-analysis study identified a correlation between psoriasis and hyperuricemia and a difference in SUAC levels between patients with psoriasis and controls in Western Europe. Future investigations are needed to establish a causal relationship between psoriasis and hyperuricemia as well as SUAC levels, and to better understand the mechanisms underlying the correlation between the 2 conditions. Moreover, additional studies should determine the potential efficacy of systemic therapies for controlling SUAC levels in psoriasis. Nevertheless, our results suggest that psoriasis patients in Western Europe should be cautioned about being at higher risk of having higher SUAC levels. Moreover, detection of increased SUAC levels in this patient population may enable earlier implementation of preventive measures for metabolic disturbances and awareness of CVD-associated risk of mortality.
